# Novel lincRNA SLINKY is a prognostic biomarker in kidney cancer

**DOI:** 10.18632/oncotarget.15703

**Published:** 2017-02-24

**Authors:** Xue Gong, Zurab Siprashvili, Okyaz Eminaga, Zhewei Shen, Yusuke Sato, Haruki Kume, Yukio Homma, Seishi Ogawa, Paul A. Khavari, Jonathan R. Pollack, James D. Brooks

**Affiliations:** ^1^ Department of Urology, School of Medicine, Stanford University, Stanford, California, USA; ^2^ Department of Pathology, School of Medicine, Stanford University, Stanford, California, USA; ^3^ Program in Epithelial Biology, School of Medicine, Stanford University, Stanford, California, USA; ^4^ Department of Urology, University Hospital Cologne, Cologne, Germany; ^5^ Department of Pathology and Tumor Biology, Graduate School of Medicine, Kyoto University, Kyoto, Japan; ^6^ Department of Urology, Graduate School of Medicine, The University of Tokyo, Tokyo, Japan

**Keywords:** lincRNA, renal cell carcinoma, kidney cancer, biomarker, prognostication

## Abstract

Clear cell renal cell carcinomas (ccRCC) show a broad range of clinical behavior, and prognostic biomarkers are needed to stratify patients for appropriate management. We sought to determine whether long intergenic non-coding RNAs (lincRNAs) might predict patient survival. Candidate prognostic lincRNAs were identified by mining The Cancer Genome Atlas (TCGA) transcriptome (RNA-seq) data on 466 ccRCC cases (randomized into discovery and validation sets) annotated for ~21,000 lncRNAs. A previously uncharacterized lincRNA, SLINKY (Survival-predictive LINcRNA in KidneY cancer), was the top-ranked prognostic lincRNA, and validated in an independent University of Tokyo cohort (*P*=0.004). In multivariable analysis, SLINKY expression predicted overall survival independent of tumor stage and grade [TCGA HR=3.5 (CI, 2.2-5.7), *P* < 0.001; Tokyo HR=8.4 (CI, 1.8-40.2), *P* = 0.007], and by decision tree, ROC and decision curve analysis, added independent prognostic value. In ccRCC cell lines, SLINKY knockdown reduced cancer cell proliferation (with cell-cycle G_1_ arrest) and induced transcriptome changes enriched for cell proliferation and survival processes. Notably, the genes affected by SLINKY knockdown in cell lines were themselves prognostic and correlated with SLINKY expression in the ccRCC patient samples. From a screen for binding partners, we identified direct binding of SLINKY to Heterogeneous Nuclear Ribonucleoprotein K (HNRNPK), whose knockdown recapitulated SLINKY knockdown phenotypes. Thus, SLINKY is a robust prognostic biomarker in ccRCC, where it functions possibly together with HNRNPK in cancer cell proliferation.

## INTRODUCTION

In the United States, kidney cancer is now the 6^th^ and 10^th^ most common cancer diagnosed in men and women, respectively [1]. Conventional or clear cell renal cell carcinoma (ccRCC) is the most common histologic subtype, with nearly 60,000 cases diagnosed per year, and accounting for 14,000 yearly deaths. Currently there are no established clinical biomarkers for ccRCC, and this lack of biomarkers poses distinct clinical challenges in managing patients with renal tumors [2]. Most RCCs are discovered on imaging studies, either incidentally or when scans are obtained for evaluation of hematuria. Once a mass is identified, diagnosis usually comes after surgical removal of the mass or the kidney, or with transcutaneous needle biopsies, which are limited in value because of their small size and the heterogeneous nature of many renal tumors that can confound definitive diagnosis. In addition, biopsies are used relatively infrequently because of perceived risks of bleeding since ccRCCs can be highly vascular tumors [3]. Approximately 20-30% of small renal masses ( < 3 cm) are benign; therefore many patients are subjected to unnecessary surgeries [4]. Even in cases where the masses are malignant, many small ccRCCs can show an indolent course and be effectively managed with active surveillance, particularly in elderly patients or patients with co-morbidities where risks of surgery might outweigh the potential mortality benefit of tumor resection. New strategies for detecting clinically aggressive renal cancers are needed [4]. Furthermore, identification of molecular pathways involved in ccRCC progression and death could provide novel insights into ccRCC biology.

Previously, we have used gene-expression profiling with cDNA microarrays to identify a set of transcripts that is highly prognostic in ccRCC [5]. However, this work was limited to approximately 27,000 transcripts mostly comprised of coding genes. With the advent of large RNA-seq datasets in normal and malignant cells and tissues, a plethora of expressed non-coding RNAs have been identified [6–8]. One such class, long non-coding RNAs (lncRNAs), are typically longer than 200 nucleotides and can be found near coding genes, which they can regulate, or in intergenic regions far from genes, where they are referred to as long intergenic non-coding RNAs (lincRNAs). Notably, lncRNAs have been identified as clinically useful disease biomarkers as they can be detected in urine, blood and other bodily fluids [9–12].

Since little has been known about the function of lincRNAs in ccRCC and their potential roles as biomarkers, we annotated RNA-seq data from TCGA and investigated whether we could discover lincRNAs associated with clinical outcome. We identified several candidates that are prognostic in ccRCC and the top candidate, SLINKY, validates in an ethnically distinct dataset of ccRCC samples and provides prognostic information independent of tumor stage and grade. Investigating its function, we found that SLINKY knockdown in ccRCC cell lines reduces cell proliferation, causes cell-cycle arrest, and alters gene expression programs related to cell growth and survival. Furthermore, SLINKY binds to the Heterogeneous Nuclear Ribonucleoprotein K, whose knockdown reproduces the effects of SLINKY knockdown on cell proliferation and altered gene expression, suggesting that SLINKY and HNRNPK likely function together to drive ccRCC cell proliferation.

## RESULTS

### Cancer-specific SLINKY lincRNA is a robust prognosticator in ccRCC

**Figure 1:**
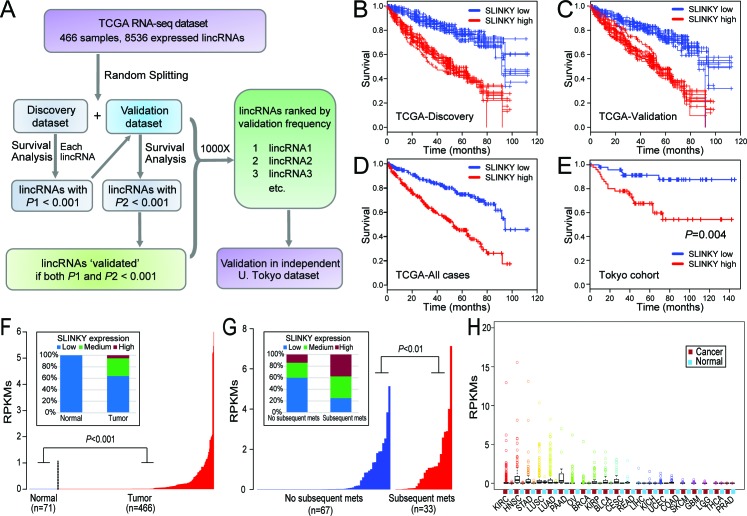
Identification and characterization of prognostic lincRNA SLINKY in ccRCC by large-scale mining of RNA-seq datasets **A**. Flowchart summarizing the approach for discovery and validation phases; see text for details. **B**.-**E**. Kaplan-Meier plots of overall survival for the top prognostic lincRNA SLINKY, shown for (B, C) ten randomly-selected iterations (of 1,000 total) from the discovery and validation phases; (D) the complete TCGA dataset; and (E) the independent Tokyo dataset. *P*-values (log-rank test) are indicated. **F**. SLINKY expression (log_2_ RPKM; ordered from lowest to highest) in normal kidney and matched ccRCC samples from the TCGA dataset; *P*-value (Mann-Whitney U-test) indicated. Inset (100% stacked column chart) summarizes SLINKY expression categorized as low (RPKM < 0.01), medium (0.01≤RPKM < 1), or high (RPKM≥1). **G**. SLINKY expression in primary ccRCC samples either without or with subsequent metastasis, from the Tokyo dataset. Inset (100% stacked column chart), as above. **H**. Box plots (median, quartiles, 2 stdev, and outliers) showing SLINKY expression across 21 different cancer types and the matched normal tissues from the TCGA dataset. N, normal; T; tumor; cancer-type acronyms are from TCGA.

To identify prognostic lincRNAs, we mined TCGA RNA-seq data on 466 ccRCC cases annotated for clinicopathologic features including patient outcome (Table 1). Although expression patterns of coding transcripts had been reported by TCGA [13], lincRNAs were not analyzed previously. We therefore enumerated lincRNA levels in each of the 466 samples by counting RNA-seq reads (RPKMs) mapping to each of ~21,000 recently annotated lncRNAs [14, 15]. In all, 8,536 lincRNAs were expressed (mean RPKM > 0.01 across the samples) in the dataset. To find candidate prognostic lincRNAs, we used a split discovery-validation sample set strategy, with multiple reiterations (summarized in Figure 1A). We randomly divided the 466 TCGA samples into equal-sized discovery and validation sets. In the discovery set, we performed Kaplan-Meier analysis (overall survival, comparing samples above and below median lincRNA expression) to identify lincRNAs with log-rank test *P* values < 0.001 (equivalent to FDR < 0.05 in 1,000 permutations, see Methods). Those lincRNAs passing the significance threshold were next tested in the validation set, and lincRNAs with *P* values < 0.001 also in the validation set were scored as ‘validated’. The randomized splitting of TCGA samples, discovery and validation steps were then repeated 1,000 times, and for each lincRNA the frequency of validation was tabulated (Supplementary Table S1).

**Table 1:** Clinicopathologic features of ccRCC cohorts

		TCGA	Tokyo	*P*-value
TOTAL (n)		466	100	
				
Sex				
	Male	307 (66%)	77 (77%)	0.03^a^
	Female	159 (34%)	23 (23%)	
				
Age (median)		61	64	0.02^b^
				
Stage	I	223 (48%)	65 (65%)	0.01^c^
	II	47 (10%)	10 (10%)	
	III	115 (25%)	13 (13%)	
	IV	81 (17%)	12 (12%)	
				
Grade	1	6 (1%)	13 (13%)	<0.01^c^
	2	197 (42%)	58 (58%)	
	3	185 (40%)	22 (22%)	
	4	72 (16%)	5 (5%)	
	Unknown	6 (1%)	0 (0%)	

^a^Fisher-exact test

^b^Mann-Whitney U-test

^c^Chi-square test

Elevated expression of the top prognostic lincRNA, ENSG00000228742 (Ensemble gene annotation), predicted significantly worse overall survival in 90% of the 1,000 random splits of the TCGA dataset. The finding was robust, as the same lincRNA emerged as the top hit across a range of *P*-value thresholds (0.0001, 0.001, 0.01, 0.05). Representative Kaplan-Meier plots are shown for the discovery and validation phases (Figure 1B, 1C), as well as for the combined dataset (Figure 1D). Based on its clinical association, we named this previously uncharacterized lincRNA “Survival-predictive LincRNA in Kidney cancer”, or “SLINKY”.

To further evaluate SLINKY, we analyzed RNA-seq data for SLINKY expression in an independent cohort of 100 ccRCC cases studied at the University of Tokyo [16]. Elevated SLINKY expression was associated with significantly worse survival in patients treated by nephrectomy (*P* = 0.004, log-rank test; Figure 1E). Of note, the Tokyo cohort included a larger proportion of patients with lower stage disease, and was ethnically distinct from the TCGA cohort, both of which might account for the better outcomes observed.

SLINKY expression appeared to be cancer specific since it was not detected (RPKM < 0.001) in normal kidney samples (Figure 1F), but was measurable (RPKM > 0.001) in 59% of tumor samples (*P* < 0.001; Mann-Whitney U-test). Interestingly, in the Tokyo cohort, SLINKY expression was significantly higher among primary tumors of patients who later (following nephrectomy) developed metastasis (*P* < 0.01, Mann-Whitney U-test; Figure 1G). SLINKY expression did not correlate with the presence of either *VHL* or *PBRM1* mutation (Supplementary Figure S1).

Since many lncRNAs exhibit tissue-specific expression patterns [15], we surveyed SLINKY expression across other normal and malignant tissue types using RNA-seq data from TCGA. Notably, SLINKY was not detected in any of 15 different normal tissue types, but was expressed in many of the corresponding cancers (Figure 1H). In particular, SLINKY expression was pronounced in subsets of head and neck squamous cell carcinoma, stomach adenocarcinoma, lung squamous cell carcinoma, lung adenocarcinoma, pancreatic adenocarcinoma, papillary renal cell carcinoma, squamous cell carcinoma of the cervix, and to a lesser degree in several others. While elevated expression was relatively common in other malignancies, SLINKY was prognostic only in head and neck squamous cell carcinoma (Supplementary Figure 2).

SLINKY expression was a significant predictor of overall survival in univariable analysis of both the TCGA and Tokyo cohorts (Table 2). In multivariable analysis, elevated SLINKY expression remained a significant predictor of overall survival in the TCGA cohort (HR = 3.5, *P* < 0.001; Table 2) and in the Tokyo dataset (HR = 8.4, *P* < 0.01), independent of tumor stage and Furman grade.

**Table 2:** Univariable and multivariable analysis

Analysis	Variable	Hazard Ratio (95%CI)	*P*-value^a^
TCGA Univariable	Sex	1.07 (0.70-1.64)	0.76
	Age (per year)	1.05 (1.03-1.06)	<0.001
	Tumor Stage II vs. I	0.95 (0.44-2.03)	0.89
	Tumor Stage III vs. I	2.79 (1.80-4.31)	<0.001
	Tumor Grade 3 vs. 1&2	1.49 (0.94-2.36)	0.09
	Tumor Grade 4 vs. 1&2	4.28 (2.35-7.77)	<0.001
	SLINKY Expression	3.58 (2.22-5.77)	<0.001
			
TCGA Multivariable	Sex	1.02 (0.65-1.60)	0.93
	Age (per year)	1.05 (1.03-1.07)	< 0.001
	Tumor Stage II vs. I	0.91 (0.42-1.97)	0.81
	Tumor Stage III vs. I	2.20 (1.38-3.51)	< 0.001
	Tumor Grade 3 vs. 1&2	1.10 (0.68-1.78)	0.71
	Tumor Grade 4 vs. 1&2	2.37 (1.26-4.45)	<0.01
	SLINKY Expression	3.53 (2.17-5.74)	< 0.001
			
Tokyo Univariable	Sex	0.50 (0.11-2.16)	0.34
	Age (per year)	1.05 (1.00-1.11)	0.06
	Tumor Stage II vs. I	3.38 (0.84-13.53)	0.09
	Tumor Stage III vs. I	7.73 (2.44-24.43)	<0.001
	Tumor Grade 2 vs. 1	1.74 (0.21-14.11)	0.61
	Tumor Grade 3&4 vs. 1	5.42 (0.67-44.16)	0.14
	SLINKY Expression	8.01 (1.80-35.67)	<0.01
			
Tokyo Multivariable	Sex	0.87 (0.17-4.49)	0.86
	Age (per year)	1.04 (0.99-1.10)	0.11
	Tumor Stage II vs. I	7.77 (1.61-37.58)	< 0.05
	Tumor Stage III vs. I	5.44 (1.64-18.12)	< 0.01
	Tumor Grade 2 vs. 1	2.49 (0.22-27.62)	0.46
	Tumor Grade 3&4 vs. 1	7.53 (0.64-88.52)	0.11
	SLINKY Expression	8.44 (1.77-40.23)	< 0.01

^a^Wald test

**Figure 2:**
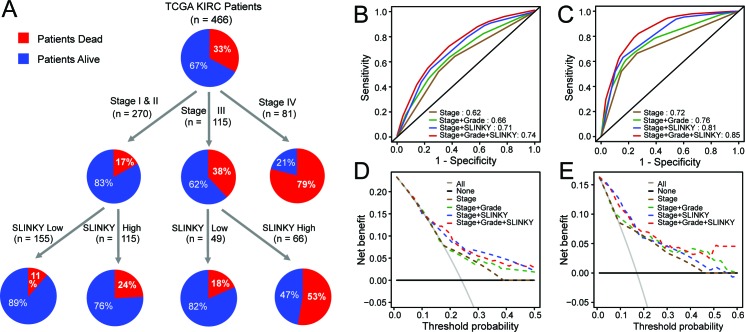
SLINKY adds prognostic information beyond clinical predictors of outcome **A**. Decision Tree analysis for the TCGA data using tumor stage, grade and SLINKY expression. **B**. Survival ROC analysis in TCGA. **C**. Survival ROC analysis for the Tokyo dataset. **D**. Decision Curve Analysis for the TCGA dataset. **E**. Decision Curve Analysis for the Tokyo dataset.

Decision tree analysis was used to define the parameters most important for patient survival prediction in the TCGA cohorts. A prediction model that included stage and SLINKY showed the highest reliability for predicting survival, and in the presence of SLINKY tumor grade did not add predictive value to the model. Stage was most predictive of outcome, and in patients with stage IV RCC, SLINKY was not significant (Figure 2A), likely because of the universally poor outcomes for those patients. When Stages I-III patients were considered, SLINKY expression significantly contributed to prediction of outcome. For example, high SLINKY expression in patients with Stage III RCC was associated with an approximate 3-fold higher death rate compared to the low expression group (53% vs. 18%, *P* < 0.001). For the stage I and II patients, high SLINKY levels were associated with more than double the risk of death (24% vs. 11%, *P* < 0.01).

ROC analysis of overall survival demonstrated that the addition of SLINKY expression to stage plus grade improved the area under curve (AUC) from 0.66 to 0.74 in the TCGA cohort. The improved prognostic accuracy was also evident in the independent Tokyo cohort, where the AUC of the ROC curve for stage plus grade improved from 0.76 to 0.85 with the addition of SLINKY (Figures 2B and 2C). Decision curve analysis confirmed that Stage + Grade + SLINKY expression had the greatest net benefit for patients compared to the other combinations, and Stage + SLINKY expression outperformed Stage + Grade for both the TCGA and Tokyo datasets (Figure 2D and 2E).

### SLINKY promotes cancer cell proliferation

**Figure 3:**
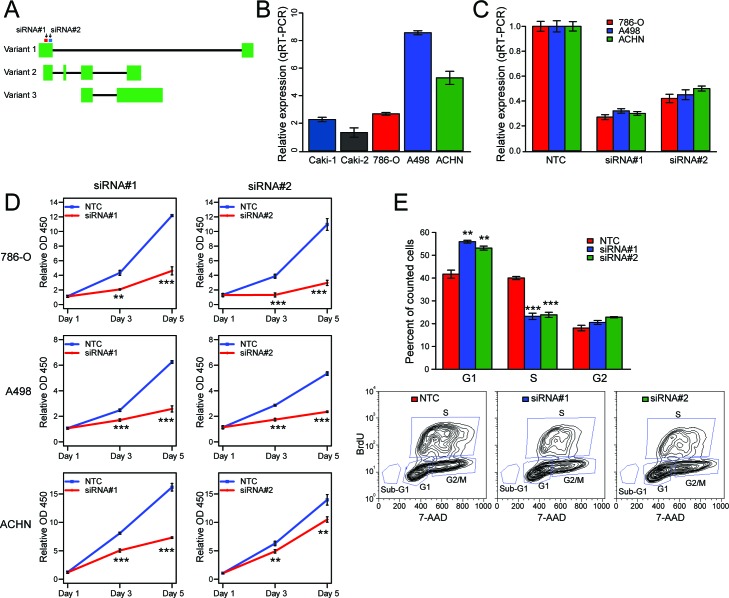
Knockdown studies reveal SLINKY role in ccRCC cell proliferation **A**. Schematic of SLINKY genomic locus illustrating exon usage of the three GENECODE-annotated transcript variants. The siRNA target sites are indicated. **B**. SLINKY expression levels in five ccRCC cell lines, quantified by Q-RT-PCR and normalized to GAPDH. **C**. Knockdown efficiency of the two siRNAs targeting SLINKY, by Q-RT-PCR 48hrs post-transfection. **D**. Cell proliferation assayed (by Wst-1 reagent) at 1, 3 and 5 days post-transfection of two different siRNAs targeting SLINKY, compared to a non-targeting control (NTC) siRNA. *P*-values (Student's t-test are indicated). **E**. Cell-cycle distribution analysis by BrdU labelling, 48 hours post-transfection of siRNAs. Below, representative FACS plots are shown. ** corresponds to a *P* value < 0.01, *** with a *P* value < 0.001.

The finding that SLINKY lincRNA was highly prognostic implies a possible mechanistic role in ccRCC development and/or progression. To investigate SLINKY function, we first examined whether knockdown of SLINKY expression in ccRCC cell lines affected cancer cell growth. Based on GENCODE v16 annotations, SLINKY is expressed as three distinct transcript variants by alternative splicing (Figure 3A). The two most highly expressed SLINKY transcript variants (from the TCGA ccRCC data) both include the first exon, and therefore we designed two different siRNAs targeting distinct sequences within that exon (Figure 3A). By Q-RT-PCR, SLINKY was expressed in all of five ccRCC cell lines we surveyed (Caki-1, Caki-2, 786-O A498 and ACHN; Figure 3B). Since the 786-O, A498 and ACHN cell lines displayed the highest SLINKY expression levels, we chose these for the knockdown studies. The two different siRNAs consistently produced approximately 70% and 50% reduction of SLINKY expression in each of the three cell lines (Figure 3C). Notably, in all three cell lines SLINKY knockdown significantly reduced cell proliferation measured at 3 and 5 days after siRNA transfection (*P* < 0.01, Student's t-test; Figure 3D).

To determine how SLINKY affected cell proliferation, we evaluated cell-cycle distributions by BrdU labeling and flow cytometry. Compared to non-targeting control cells, SLINKY knockdown reduced the percentage of cells in S phase (DNA synthesis, *P* < 0.001; Figure 3E), with a corresponding increase of cells in G_0_/G_1_ (*P* < 0.01), consistent with G_1_ arrest. The percentage of sub-G_0_/G_1_ (apoptotic) cells was similar between the control and SLINKY knockdown cells, indicating little or no induction of apoptosis. Using a Matrigel cell invasion assay, we observed that SLINKY knockdown did not affect cell invasion (Supplementary Figure S3).

### SLINKY impacts cell proliferation and cancer pathways

Several lincRNAs have been found to regulate the expression of coding genes immediately adjacent on the chromosome [17]. SLINKY resides about 60 Kb upstream of *PIK3CG* (Phosphatidlyinositol-4,5-Bisphosphate 3-Kinase, Catalytic Subunit Gamma) on chromosome 7 (Supplementary Figure S4A). Because the PI3K pathway is a known cancer pathway and one with relevance in ccRCC [18], we tested whether SLINKY knockdown affected PIK3CG expression. However, SLINKY knockdown did not alter PIK3CG expression levels (which were not detectable at baseline), measured by RNA-seq (Supplementary Figure 4B).

**Figure 4:**
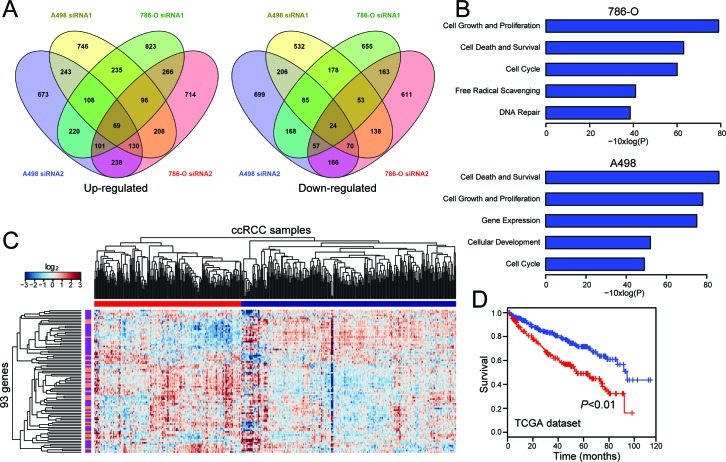
Transcriptome (RNA-seq) analysis of SLINKY knockdown identifies gene signatures relevant to proliferation and prognostic in tumor specimens **A**. Peacock plots illustrate genes ≥1.25-fold upregulated (left) or downregulated (right) following SLINKY knockdown in each of two ccRCC cell lines. **B**. Significantly enriched biological functions (by Ingenuity Pathway Analysis) associated with SLINKY knockdown in each of the two cell lines. **C**. Hierarchical clustering of TCGA samples across the 93 genes affected by SLINKY knockdown (common among both cell lines and both siRNAs). Note, two main sample clusters are observed (red and blue bars), where the cluster (red) with higher SLINKY expression shows significant enrichment of genes (purple bars) downregulated with SLINKY knockdown (*P* < 0.01, Fisher's Exact Test). **D**. Kaplan-Meier survival analysis comparing the two TCGA sample clusters from above; *P*-value (log-rank test) indicated.

To better understand the role of SLINKY in ccRCC cells, we assayed whole-transcriptome changes (by RNA-seq) following SLINKY knockdown in 786-O and A-498 cells, using two different SLINKY siRNAs compared to non-targeting control. In each cell line, several hundred transcripts were either up- or down-regulated (≥1.25-fold) upon SLINKY knockdown, with significant overlaps between the two siRNAs (*P* < 0.001, hypergeometric test; Figure 4A). A smaller (but still significant) subset of up- and down-regulated transcripts (n = 93, Supplementary Table S2) was shared among both cell lines and both siRNAs (*P* < 0.001) (Figure 4A). In both cell lines, the altered transcripts common to both siRNAs showed significant enrichment for cell cycle, cell proliferation and survival functions by Ingenuity Pathway Analysis (Figure 4B). The pathway analysis results were consistent with the observed reduced cell proliferation noted above.

We also sought to determine whether the transcriptome changes observed (upon SLINKY knockdown) in ccRCC cell lines were relevant to patient tumor samples. If so, we might expect those genes whose expression was altered upon SLINKY knockdown in cultured ccRCC cells to correlate with SLINKY expression in tumors. Using the TCGA dataset, we identified 381 genes whose expression levels correlated with SLINKY expression in ccRCC tumor samples (Pearson correlation ≥ 0.15). Comparing this list with the list of genes showing altered expression upon SLINKY knockdown in each ccRCC cell line, we observed a significant overlap (*P* < 0.01; Hypergeometric test).

In a complementary approach, we used the 93 genes showing altered expression upon SLINKY knockdown in both ccRCC cell lines to cluster the TCGA patient samples. The ccRCC samples clustered into two major groups (Figure 4C) that were associated with significantly different patient survival (*P* < 0.01; log-rank test; Figure 4D). Notably, the worse outcome group was significantly enriched for cases with elevated SLINKY expression (*P* < 0.01; Fisher's exact test). Also noteworthy, the worse-outcome group was associated with a gene cluster significantly enriched for genes downregulated with SLINKY knockdown (*P* < 0.01; Fisher-exact test), while the better-outcome group showed the converse (*P* < 0.01). Thus, the gene expression patterns from SLINKY knockdown in cultured ccRCC cells demonstrate a striking relevance to SLINKY expression in patient tumor samples.

### SLINKY may function through binding Heterogeneous Nuclear Ribonucleoprotein K

**Figure 5:**
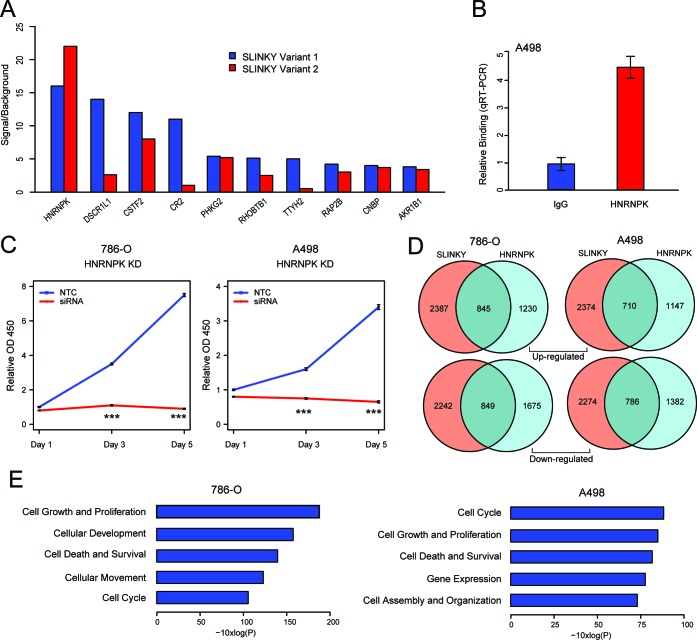
SLINKY binds to HNRNPK, an interaction likely promoting cell proliferation **A**. Top 10 protein binding interactions of SLINKY transcript variants 1 and 2, identified by hybridization to a human recombinant protein microarray. Proteins are ordered by binding intensity (fluorescence signal to background). **B**. Confirmation of SLINKY-HNRNPK interaction in A498 cells by RNA immunoprecipitation, using an anti-HNRNPK antibody. SLINKY binding fold-enrichment over IgG control is shown. **C**. Cell proliferation assayed (by WST-1 reagent) at 1, 3 and 5 days post-transfection of an siRNA pool targeting HNRNPK, compared to non-targeting control (NTC) siRNA. ***, *P* value < 0.001. **D**. Venn diagrams illustrate gene numbers upregulated (top) or downregulated (bottom) ≥1.25-fold following SLINKY and HNRNPK knockdown in each of two ccRCC cell lines. **E**. Significantly enriched biological functions (by Ingenuity Pathway Analysis) associated with the genes whose expression is altered by both SLINKY and HNRNPK knockdown, shown for each of the two ccRCC cell lines.

LncRNAs often function through interaction with specific proteins or protein complexes [19]. To discover potential binding partners of SLINKY, we carried out an *in vitro* binding screen of fluorescently labeled SLINKY transcript against 9,125 different recombinant human proteins in high-density microarray format [20]. Assayed separately, SLINKY transcript variants 1 and 2 (the most abundant isoforms; Figure 3A) bound a small, mostly common set of the arrayed proteins (Figure 5A). The top binding partner, Heterogeneous Nuclear Ribonucleoprotein K (HNRNPK), displayed a 15-fold binding signal above background. We confirmed the binding of SLINKY to HNRNPK in living cells (A498 ccRCC cells) by native RNA immunoprecipitation (RIP) (4.3-fold enrichment; Figure 5B).

To evaluate the functional relevance of the SLINKY-HNRNPK interaction, we used siRNA to knockdown HNRNPK in the same ccRCC cell lines previously assessed for SLINKY knockdown. Notably, HNRNPK knockdown led to reduced cell proliferation, at least comparable with SLINKY knockdown (Figure 5C). Moreover, the transcriptome changes induced by HNRNPK knockdown displayed a marked overlap (about one-third) with those observed with SLINKY knockdown, significantly more than expected by chance (*P* < 0.001; hypergeometric test; Figure 5D). Furthermore, in both cell lines, the altered genes common to SLINKY and HNRNPK knockdown exhibited significant enrichment for cell proliferation, cell survival and cell cycle functions by Ingenuity Pathway Analysis (Figure 5E). Taken together, the binding studies and the overlap of knockdown phenotypes suggest that SLINKY directly interacts with HNRNPK to effectuate enhanced ccRCC cell proliferation.

## DISCUSSION

We have identified SLINKY as a potentially significant lincRNA in ccRCC biology that could serve as an important biomarker of ccRCC prognosis. Elevated expression of SLINKY was a strong predictor of worse overall survival in the TCGA cohort, and validated in a smaller set of ccRCCs in an ethnically distinct cohort of patients from Japan. Furthermore, based on our analysis of additional TCGA datasets, SLINKY is over-expressed in other tumor types and is a candidate prognostic marker in squamous cell carcinomas of the head and neck. SLINKY appears to regulate cell proliferation but not invasion in ccRCC cells in culture by modulating genes associated with the cell cycle. The genes regulated by SLINKY in cell culture appear to be relevant to SLINKY activities *in vivo*, since the gene set both correlates with SLINKY expression in tumors and with clinical outcome in the TCGA dataset. Mechanistically, binding and knockdown studies suggest that interaction between SLINKY and the heterogeneous nuclear ribonucleoprotein HNRNPK underlies the effect of SLINKY on cell proliferation. Taken together, SLINKY appears to be a novel lincRNA that is upregulated across several malignancies and that drives aggressiveness in ccRCC by regulating cancer cell proliferation.

The role of lincRNAs in carcinogenesis and cancer progression, including in ccRCC, is only beginning to emerge. One of the most well characterized lincRNAs, HOTAIR, was identified in primary and metastatic breast cancers and its expression level is a powerful predictor of patient survival [21]. HOTAIR is over-expressed in several RCC cell lines and knockdown of HOTAIR inhibits cell proliferation [22]. The lincRNAs MALAT-1and NBAT-1 have been identified in several malignancies and alteration of their expression levels has been shown to correlate with adverse outcomes in ccRCC [23, 24]. While additional candidate lincRNAs have been investigated in ccRCC, such as H19 and MEG3, very few unbiased assessments of the roles of lincRNAs in ccRCC have been undertaken [25]. Studies involving relatively small numbers of normal and malignant kidney tissues have identified novel candidate lncRNAs that are altered in ccRCC, but have been underpowered to detect robust biomarkers of prognosis [26, 27]. Using TCGA data, Malouf *et al*. identified 1,934 lincRNAs that clustered ccRCC into four discrete groups that differed in the spectrum of mutation of known drivers of RCC as well as differences in DNA copy number alterations [28]. One of the four groups, comprising 111/475 cases, showed significantly worse prognosis. Our study is unique in focusing on identifying individual “driver” lncRNAs that are correlated with prognosis, and in our validation in an independent, ethnically distinct dataset.

Several mechanisms have been identified by which lincRNAs contribute to cancer genesis and progression [19]. Many lincRNAs are found in the nucleus where they affect gene expression by direct or indirect interactions with chromatin remodeling complexes. For example, HOTAIR reprograms chromatin state to promote cancer metastasis by redirecting the Polycomb Repressive Complex 2 (PRC2) to active genes involved in embryogenesis while suppressing a set of genes that block metastases [21]. Similarly SChLAP1, a lincRNA over-expressed in aggressive prostate cancer, interferes with the localization of the chromatin binding SWI/SNF complex [29].

In our study, we find that SLINKY enhances cancer cell proliferation most likely through its interaction with HNRNPK. A member of the heterogeneous nuclear ribonucleoprotein family, HNRNPK has varied functions in regulating transcription (either directly, or indirectly through binding chromatin modifying complexes) and protein translation, and has been implicated as an oncogene or tumor suppressor in diverse human malignancies [30, 31]. Notably, HNRNPK has recently been reported to bind other lncRNAs, including Xist [32], lincRNA-p21 [33], and EWSAT1 [34]. In those instances, it has been demonstrated or inferred that the lncRNA serves to recruit HNRNPK to specific genomic loci to affect gene silencing. In our studies, knockdown of either SLINKY or HNRNPK leads to both the upregulation and downregulation of genes, so the possible recruitment of HNRNPK to gene loci (yet to be proven) might serve to either activate or repress genes. Regardless, our study along with others suggests that lncRNA recruitment of HNRNPK might serve as a generalized mechanism to affect the gene expression changes that drive cancer. Whether SLINKY binds to other proteins (either identified on the protein microarray or yet to be discovered) to affect its biological functions remains to be determined.

Irrespective of its biologic role, SLINKY is an excellent candidate biomarker of prognosis in ccRCC. One significant challenge in identifying robust biomarkers is the failure to reproduce in independent cohorts, likely due to weak predictive value of the biomarkers, unique features of the discovery cohort, and statistical methodologies used. We used a unique process to identify candidate biomarkers by random splits of the large TCGA cohort into discovery and validation sets, with multiple (1,000x) iterations. By capturing the number of times each candidate lincRNA was validated, we identified SLINKY as our top candidate and were able to validate its ability to predict survival in a cohort that was ethnically distinct, with data produced in an independent laboratory, and in patients with on average lower stage. SLINKY expression appears to be confined to cancerous tissues and is not observed in normal tissues; therefore, there should be little or no contamination of measurements of SLINKY in target tissues or bodily fluids. This opens the possibility of designing urine-based or blood-based assays for SLINKY.

There are notable precedents for using lincRNAs as clinical biomarkers. For example, the Prostate Cancer Associated gene 3 (PCA3) lncRNA is highly expressed in prostate cancer and can be detected in the urine of patients with prostate cancer [12]. PCA3 is an FDA-approved assay used to select patients at risk for prostate cancer who need repeat prostate biopsies after a prior negative biopsy. Similarly, clinical assays for SChLAP1 tissue expression have been developed and shown to be effective at identifying future metastases in patients treated for localized prostate cancer in a multi-institutional trial [35]. While additional work will be necessary to develop assays for SLINKY and validate their performance in predicting disease outcomes, there are significant clinical needs in identifying at risk patients on surveillance for small renal lesions, and for patients undergoing nephrectomy who might need more intense clinical follow-up or adjuvant therapy.

## MATERIALS AND METHODS

### Patient cohorts and RNA-seq datasets

The TCGA cohort comprises 466 ccRCC cases; clinicopathologic features are summarized in Table 1. The independent validation dataset (University of Tokyo) comprises 100 cases (Table 1). RNA-seq data (BAM files) with associated de-identified clinical data from TCGA were obtained from the Cancer Genomics Hub (CGHub, https://cghub.ucsc.edu/) and TCGA Data Portal (https://gdc.cancer.gov/) with approved authorization from TCGA. For the validation cohort, comparable RNA-seq data (BAM files) with associated de-identified clinical data from a previous study of ccRCC in Japanese patients were processed in an identical fashion. As detailed previously, those tumor samples and clinical data were obtained under institutional review board approval and with patient informed consent [16]. For select other cancer types, RNA-seq data (with corresponding lincRNA annotations) were retrieved from MiTranscriptome compendium [6].

### LincRNA annotation and expression

Annotations for human lncRNAs were collected from both GENCODE v16 [14] and the predictions assembled from RNA-seq data of 24 tissues and cell types [15]. When transcripts overlapped between the two data sources, the GENCODE annotations were used. Our analysis focused exclusively on ~21,000 annotations of long non-coding RNAs (lncRNAs). Cufflinks [36] was used to calculate RPKMs (reads per kilobase per million mapped reads) as a means of quantifying the expression of the lincRNAs from the BAM files. Subsequent analysis focused on the 8,536 lincRNAs that were expressed in the TCGA ccRCCA samples as defined by mean RPKM > 0.01 across the samples.

### Identification of prognostic lincRNAs

TCGA ccRCC samples were randomly split into equal-sized discovery and validation sets, and Kaplan-Meier analysis was performed for each lincRNA in the discovery set (overall survival, comparing samples above and below the median lincRNA expression). LincRNAs that predicted survival, based on a log-rank test *P*-value < 0.001, were passed onto the validation set. Those lincRNAs with a log-rank test *P*-value < 0.001 also in the validation set were scored as validated. (To evaluate the robustness of the lincRNAs identified in TCGA, we also tested different *P* value thresholds including 0.0001, 0.001, 0.01, and 0.05.) The random splitting of TCGA samples into discovery and validation sets was then repeated 1,000 times and for each lincRNA the frequency of validation was tabulated such that lincRNAs could be ranked by their validation frequency. In a parallel analysis, ccRCC sample labels were first randomly permuted (i.e. dissociating lincRNA expression profiles from clinical data) and the same process was repeated 1,000 times to obtain a null distribution of validation frequencies, permitting estimation of a false discovery rate (FDR) for multiple test correction. Kaplan-Meier, multivariable Cox Proportional Hazards, and survival Receiver Operating Characteristic (ROC) curve analyses were performed using R. Decision tree analysis was done by the Chi-square Automatic Interaction Detection (CHAID) method, using SPSS (version 23). The relative value of the prognostic models was assessed by decision curve analysis [37] using R.

### Cell culture

Clear cell renal cell carcinoma cell lines were obtained from the American Type Culture Collection (ATCC), where cell line authentication was done by Short Tandem Repeat profiling. A498 and ACHN cells were cultured in DMEM, Caki-1 and Caki-2 in McCoy's medium, and 786-O in RPMI-1640 , all supplemented 10% (vol/vol), fetal bovine serum, L-glutamate, and Pen/Strep.

### Q-RT-PCR

Total RNA was isolated from ccRCC cell lines (Qiagen, RNeasy) and reverse transcribed using SuperScript II (Life Technologies). SLINKY transcript levels were quantified by Q-PCR using SYBR Green reagents (Applied Biosystems) and gene-specific primers (Supplementary Table S3) on an ABI 7500 Fast System. Assays were done in triplicate, and relative transcript levels calculated by the CT method and normalized to GAPDH.

### siRNA transfections and proliferation assays

ccRCC cells were seeded (10,000 cells per well) in 6-well plates and transfected using Lipofectamine 2000 (Life Technologies). Two independent small interfering (si)RNAs targeting the first exon of SLINKY (On-TARGETplus, Dharmacon) were transfected at a final siRNA concentration of 50nM for 16 hrs (custom-designed siRNA sequences available in Supplementary Table S3). SiRNA targeting HNRNPK comprised a catalog ON-TARGETplus SMARTpool (Dharmacon). Non-targeting control (NTC) siRNA (ON-TARGETplus Non-targeting Control Pool; Dharmacon) was used for comparison. Cell proliferation/viability was then quantified 1, 3 and 5 days post-transfection by WST-1 assay (Roche). All assays were done in triplicate and significant differences assessed by Student's t-test.

### BrdU flow cytometry

Cell-cycle distribution was evaluated using the FITC BrdU Flow Kit (BD Biosciences). Bromodeoxyuridine (BrdU) was added to cells for 1 hr, and cells were then collected, fixed, permeabilized and stained with FITC-BrdU antibody and 7-AAD. BrdU and 7-AAD profiles were collected on a BD FACSCalibur using CellQuest software (BD Biosciences) and analyzed using FlowJo software (Treestar).

### Invasion assays

Invasion assays were done using BioCoat Matrigel Invasion Chambers (BD Biosciences). 48 hrs following siRNA transfection, cells were seeded in serum-free DMEM media (20,000 cells/chamber), with 10% FBS in the lower chamber serving as a chemoattractant. 18 hrs later, invaded cells were fixed with 100% methanol and stained with 0.5% crystal violet. Cells traversing the Matrigel were counted by light microscopy in five representative 10X fields. All assays were done in triplicate, and significant differences evaluated by Student's t-test.

### RNA-seq library preparation, sequencing and data analysis

ccRCC cells were transfected separately with two different siRNAs targeting SLINKY or non-targeting control (NTC) siRNA, and then total RNA was isolated 24 hrs post-transfection. Barcoded RNA-seq libraries were then prepared from total RNA using the Illumina TruSeq RNA Sample Prep Kit (v2), and sequenced (single-end, 36-bp reads) on an Illumina HiSeq 2000 instrument to a depth of approximately 50 million reads per sample. Sequence reads were mapped to the human genome (hg19) and transcripts quantified as RPKMs using Cufflinks according to RefSeq annotation. Biologic pathways implicated by gene-expression changes were identified using Ingenuity Pathway Analysis (Qiagen). The complete dataset of raw RNA-seq reads is available at GEO (Accession GSE70602). Hierarchical clustering was done in R, using average linkage clustering with Pearson correlation on mean-centered log_2_ RPKMs.

### RNA hybridization on protein microarrays

DNAs encoding SLINKY transcript variants 1 and 2 were generated by GeneArt gene synthesis (Thermo Fisher Scientific ), and subcloned into pBluescriptKS. SLINKY RNAs were then synthesized by *in vitro* transcription (Promega), and labeled with Cy5 (Label IT μArray labeling kit; Mirus) to achieve approximately 1 to 3 Cy5 fluorescence dye per transcript. Labeled RNAs were then hybridized to the ProtoArray Human Protein Microarray v5.0 (Life Technologies), and signal scanned and quantified as previously described [20]. Promiscuous RNA binding proteins, those that have bound to more than 75% of all labelled RNAs (over 50) that we have collectively assayed (ref [20] and unpublished), were excluded from analysis.

### RNA immunoprecipitation

RNA immunoprecipitations (RIP) were done following an Abcam protocol [38]. Briefly, ccRCC cells were collected, and nuclei isolated and then lysed in RIP buffer. Chromatin was sheared using a Bioruptor sonicator (Diagenode) set for four 30s cycles. HNRNPK was immunoprecipitated with a mouse anti-HNRNPK antibody (Abcam; 3C2), in comparison to normal mouse IgG control (Santa Cruz Biotechnology). Co-immunoprecipitated SLINKY transcript was then detected and quantified by Q-RT-PCR, as detailed above.

### Statistics (summary)

The association between lincRNA expression and overall survival was evaluated by Kaplan-Meier survival analysis and multivariable Cox regression analysis. The clinical utility of SLINKY was assessed by decision tree, decision curve analysis and ROC analysis for survival. The data from qRT-PCR, cell proliferation, cell-cycle and cell invasion experiments were analyzed by Student's t-test. Overlaps of transcriptome changes were assessed by hypergeometric test, and enriched biological functions/processes by Ingenuity Pathway Analysis.

## SUPPLEMENTARY MATERIALS FIGURES AND TABLES


