# Supplementary material for: Novel lincRNA SLINKY is a prognostic biomarker in kidney cancer
Source: Oncotarget. 2017 Feb 24;8(12):18657–69. doi: 10.18632/oncotarget.15703 (PMC5386637; doi:10.18632/oncotarget.15703)
Supplement: Supplementary file 1 [file oncotarget-08-18657-s001.pdf]

Novel lincRNA SLINKY is a prognostic biomarker in kidney cancer

Supplementary Material

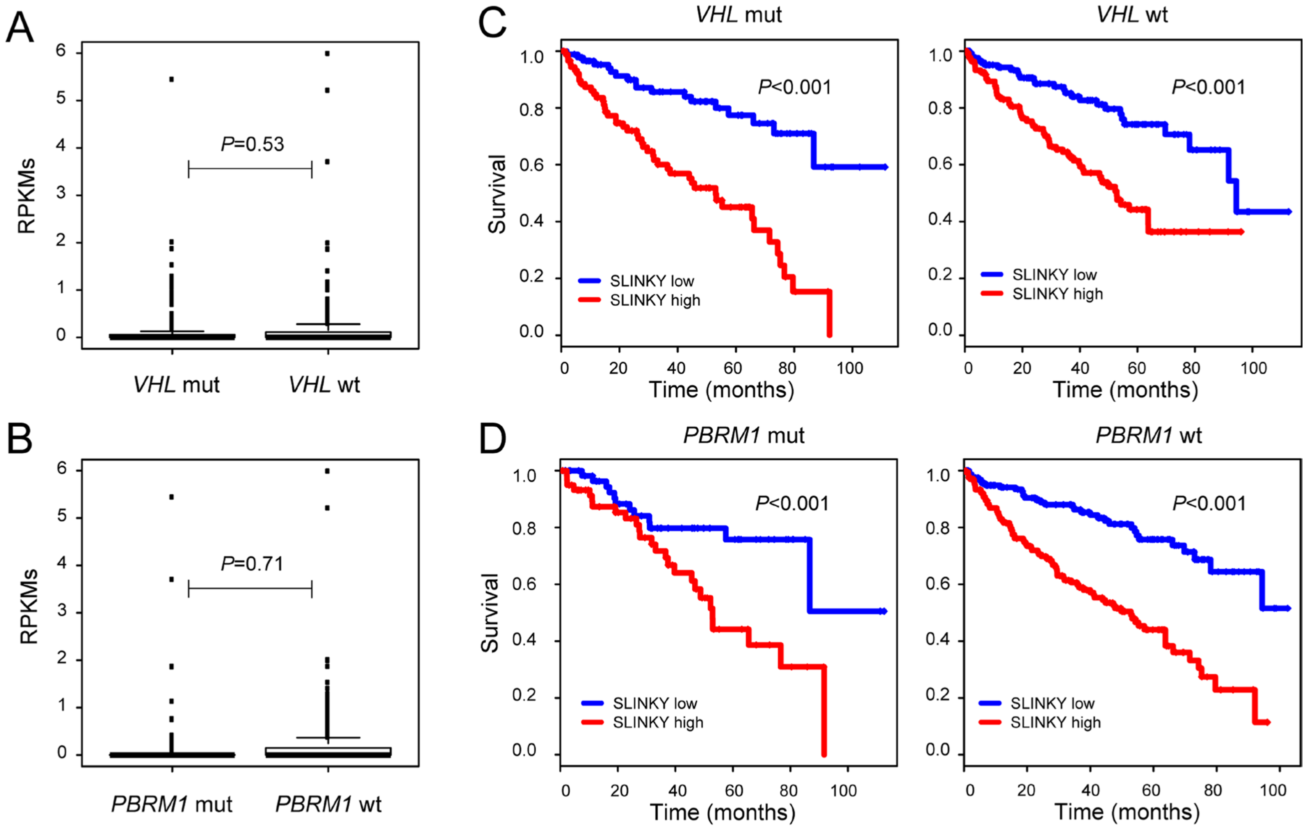

Supplementary Figure S1. SLINKY expression does not correlate with the presence of either *VHL* or *PBRM1* mutation. (A) SLINKY expression in *VHL* mutant and wildtype patients, *P*-value (Student's t-test) indicated. (B) SLINKY expression in *PBRM1* mutant and wildtype patients, *P*-value (Student's t-test) indicated. (C) Kaplan-Meier plot compares samples above and below median expression for *VHL* mutant and wildtype patients. *P*-value (log-rank test) indicated. (D) Kaplan-Meier plot compares samples above and below median expression for *PBRM1* mutant and wildtype patients. *P*-value (log-rank test) indicated.

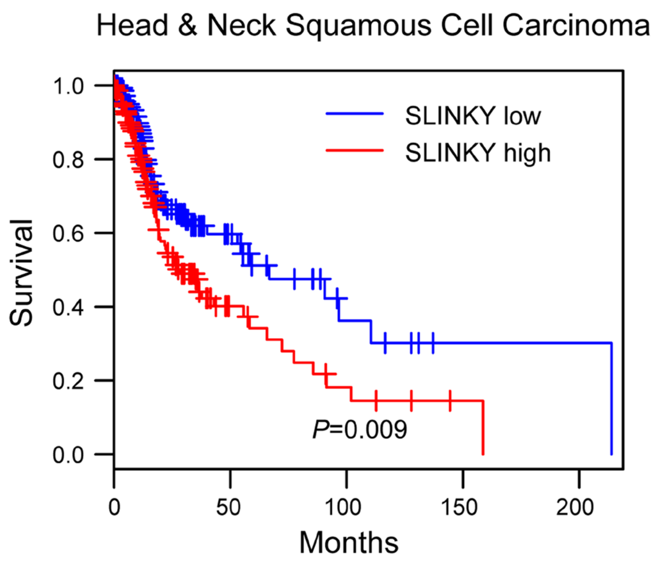

Supplementary Figure S2. SLINKY expression is prognostic in HNSCC. Kaplan Meier plot compares samples above and below median expression. *P*-value (log-rank test) indicated.

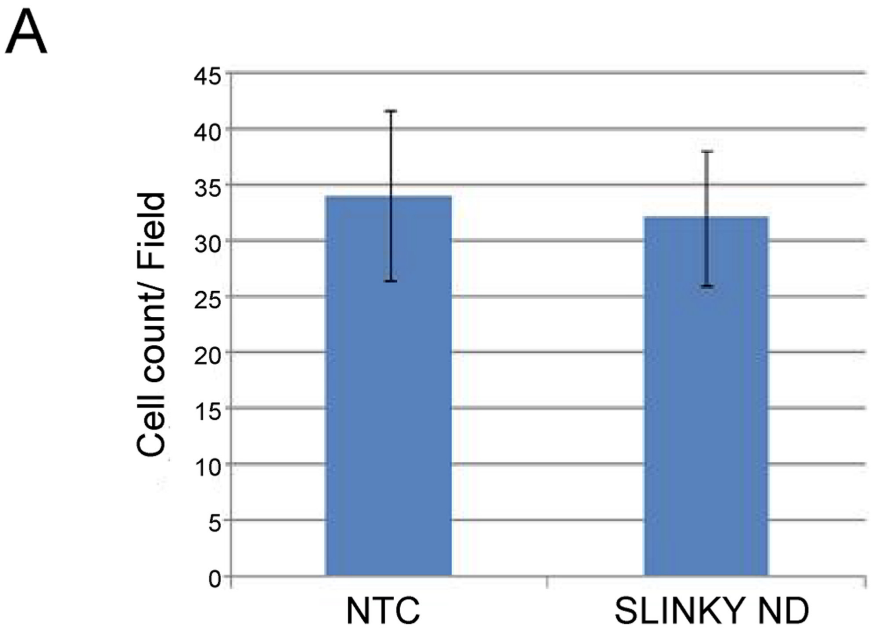

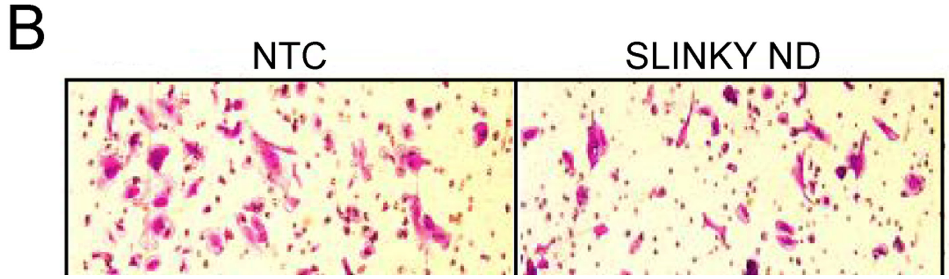

Supplementary Figure S3. SLINKY knockdown does not impact cell invasiveness. (A) Quantification of A498 cell invasion following SLINKY knockdown, compared to non-targeting control (NTC). *P*-value not significant (Mann-Whitney U-test). (B) Representative microscopic fields showing cells having invaded through the Matrigel at 48 hrs (10x microscope objective).

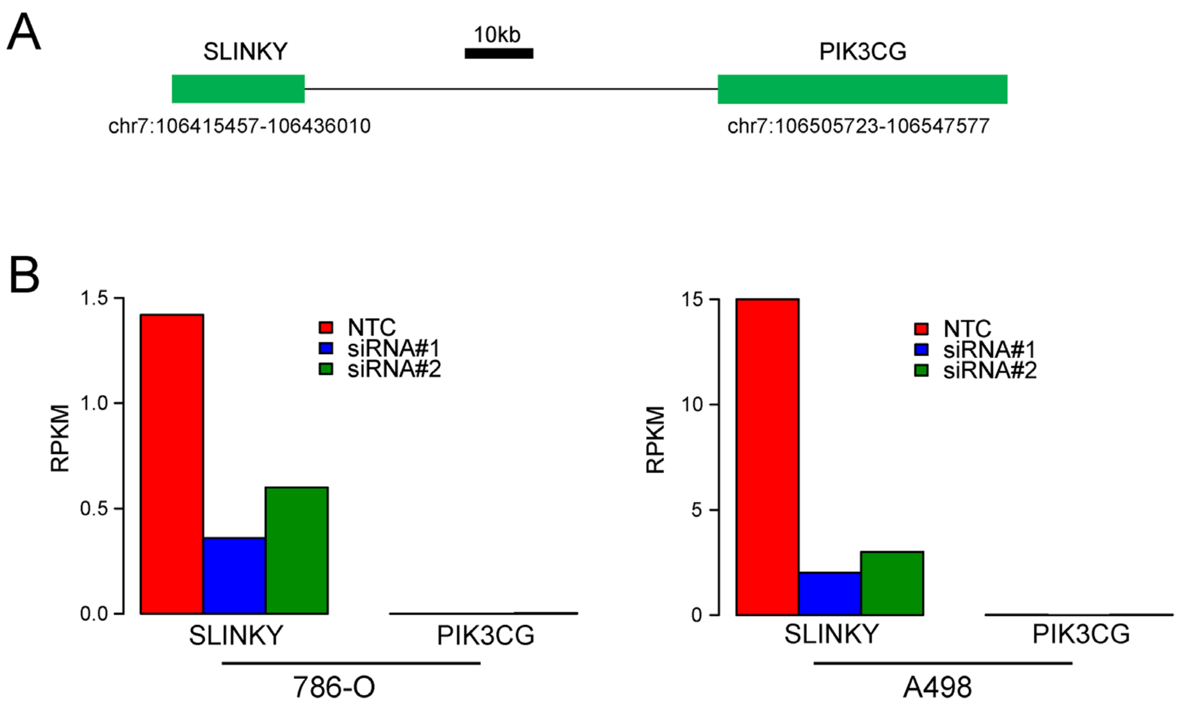

Supplementary Figure S4. SLINKY knockdown does not affect expression of chromosomal gene neighbor PIK3CG. (A) Schematic of chromosome 7 genomic locus showing SLINKY and PIK3CG. (B) PIK3CG expression levels by RNA-Seq following SLINKY knockdown, compared to non-targeting control (NTC).

Supplementary Table 1: Top prognostic lincRNAs from the TCGA dataset

LincRNA Name	Validation Frequency[a]	*P*-value[b] TCGA dataset	*P*-value[b] Tokyo dataset	Chromosome Location
SLINKY (RP5-884M6.1)	0.9	2.60E-07	0.004	chr7:106415457-106436010
LINC00524	0.71	1.11E-06	0.19	chr14:101872324-101874259
RP11-394O9.1	0.53	4.17E-05	0.09	chr9:82645494-82649470
PDZK1P1	0.52	2.54E-05	0.16	chr1:145924388-145942619
SNORD3C	0.47	2.45E-04	0.33	chr17:19092978-19093558
AP000439.3	0.36	1.03E-05	0.013	chr11:69291901-69294708
LOC100129046	0.32	1.92E-05	0.11	chr1:94057525-94065587
SNORD3B-1	0.31	4.45E-04	0.99	chr17:18965225-18965807
SNORD3A	0.25	2.92E-04	0.024	chr17:19091329-19092027
ANKRD30BL	0.19	8.27E-05	0.0088	chr2:132905164-133015542

[a]Validation frequency over the 1,000 splits of the TCGA dataset
[b]Log-rank test

Supplementary Table S2. Up- and down-regulated transcripts shared among both cell lines and both siRNAs

Up-/Down-Regulated	Gene Symbol
Up-Regulated Genes	ACYP1,AMZ2,ANAPC11,ATXN7L3,AURKA,BIRC3,BTN3A2,C17orf62,CCNE2,CEP78,CHD3,CKLF,COG5,COL4A3BP,CPT1B,CXorf40A,DDX54,DEDD,FAM195B,FBXL2,FDPS,FKBP11,GOSR1,GSTA4,GTF2H3,HBS1L,HOMER2,LDLR,MAPKAPK5,MTERFD3,NEK6,NFYC,OGG1,P2RX4,PDLIM5,QRFP,RBFOX2,RBM24,RNF145,RPS3A,SCML1,SNX12,SPAG9,SPATA20,SPP1,SRSF11,STK36,STX4,TARS2,TDG,TLE3,TMEM14C,TMEM189,TMEM230,TMEM64,TOMM5,TPM2,TPM4,TRIM33,TRIQK,TSPAN2,TXNRD1,URI1,VCAM1,WDR26,ZC3H14,ZFAND5,ZNF260,ZNF644
Up-Regulated Genes	ALKBH2,ARFIP2,ASCC1,CARS,CECR5,DHPS,GBAS,GIPC1,H2AFY,IL17RC,LSR,MVK,NOL3,NUDT1,PMEPA1,PRPSAP2,PSMG4,PTHLH,RPS6KA4,SAP30L,SPHK2,STRA13,TCEB1,TST

Supplementary Table 3: siRNA and Q-RT-PCR primer sequences

**siRNA sequences (SLINKY)**	
siRNA #1	Sense: 5'-GCCUUAACCUCCUGGAAUGUU<br>Antisense: 5'- CAUUCCAGGAGGUUAAGGCUU
siRNA #2	Sense: 5'-GGACAGCUUCUUUACGCAUUU<br>Antisense: 5'-AUGCGUAAAGAAGCUGUCCUU
**Q-RT-PCR primers**	
F	5’-TGGGTTTTGGACAGCTTCTT
R	5’-TGCATTCCAGGAGGTTAAGG
